# Oncological Outcomes of Nipple-Sparing Mastectomy in an Unselected Population Evaluated in a Single Center

**DOI:** 10.1055/s-0042-1751286

**Published:** 2022-12-29

**Authors:** Francisco Pimentel Cavalcante, Mirella Macedo Parente Araújo, Igor Moreira Veras, Ruffo Freitas-Junior

**Affiliations:** 1 Hospital Geral de Fortaleza, Fortaleza, CE, Brazil; 2 Grupo de Educação e Estudos Oncológicos, Fortaleza, CE, Brazil; 3 Centro Regional Integrado de Oncologia, Fortaleza, CE, Brazil; 4 Department of Obstetrics and Gynecology, Universidade Federal de Goiás, Goiânia, GO, Brazil

**Keywords:** mastectomy, nipple-sparing mastectomy, breast neoplasms, subcutaneous mastectomy, segmental mastectomy, mastectomia, mastectomia poupadora do complexo areolo-mamilar, neoplasias mamárias, mastectomia subcutânea, mastectomia segmentar

## Abstract

**Objective** Nipple-sparing mastectomy (NSM) has been traditionally used in selected cases with tumor-to-nipple distance > 2 cm and negative frozen section of the base of the nipple. Recommending NSM in unselected populations remains controversial. The present study evaluated the oncological outcomes of patients submitted to NSM in an unselected population seen at a single center.

**Methods** This retrospective cohort study included unselected patients with invasive carcinoma or ductal carcinoma in situ (DCIS) who underwent NSM in 2010 to 2020. The endpoints were locoregional recurrence, disease-free survival (DFS), and overall survival (OS), irrespective of tumor size or tumor-to-nipple distance.

**Results** Seventy-six patients (mean age 46.1 years) (58 invasive carcinomas/18 DCIS) were included. The most invasive carcinomas were hormone-positive (60%) (HER2 overexpression: 24%; triple-negative: 16%), while 39% of DCIS were high-grade. Invasive carcinomas were T2 in 66% of cases, with axillary metastases in 38%. Surgical margins were all negative. All patients with invasive carcinoma received systemic treatment and 38% underwent radiotherapy. After a mean of 34.8 months, 3 patients with invasive carcinoma (5.1%) and 1 with DCIS (5.5%) had local recurrence. Two patients had distant metastasis and died during follow-up. The 5-year OS and DFS rates for invasive carcinoma were 98% and 83%, respectively.

**Conclusion** In unselected cases, the 5-year oncological outcomes following NSM were found to be acceptable and comparable to previous reports. Further studies are required.

## Resumo

**Objetivo** A mastectomia poupadora do complexo areolo-mamilar (MPM) tem sido tradicionalmente utilizada em casos selecionados com distância tumor-mamilo > 2 cm e biópsia de congelação da base do mamilo negativa. Recomendar MPM em populações não selecionadas continua controverso. Este estudo avaliou os resultados oncológicos de pacientes submetidas à MPM em uma população não selecionada atendida em um único centro.

**Métodos** Coorte retrospectivo incluindo pacientes não selecionadas com carcinoma invasivo ou carcinoma ductal in situ (CDIS) submetidas à MPM entre 2010 e 2020. Os desfechos incluíram: recorrência locorregional, sobrevida livre de doença (SLD) e sobrevida global (SG), independentemente do tamanho do tumor ou da distância tumor-mamilo.

**Resultados** Setenta e seis pacientes (média: 46,1 anos de idade) (58 carcinomas invasivos/18 CDIS) foram incluídas. A maioria dos carcinomas invasivos era hormônio-positivo (60%) (superexpressão de HER2: 24%; triplo-negativo: 16%), enquanto 39% dos CDIS eram de alto grau histológico. Os carcinomas invasivos foram T2 em 66% dos casos, com metástases axilares em 38%. As margens cirúrgicas foram todas negativas. Todas as pacientes com carcinoma invasivo receberam tratamento sistêmico e 38% receberam radioterapia. Após um período médio de 34,8 meses, 3 pacientes com carcinoma invasivo (5,1%) e 1 com CDIS (5,5%) apresentaram recidiva local. Durante o acompanhamento, duas pacientes tiveram metástase à distância e vieram a óbito. As taxas de SG e SLD aos 5 anos para carcinoma invasivo foram de 98% e 83%, respectivamente.

**Conclusão** Em casos não selecionados, os resultados oncológicos de 5 anos após MPM foram considerados aceitáveis e comparáveis a resultados anteriores. Estudos adicionais são necessários.

## Introduction

The surgical treatment of early breast cancer has progressed significantly over recent decades. The concept of radical mastectomy has gradually given way to the development of breast-conserving surgery. Evidence from several randomized clinical trials shows that the survival rates after breast-conserving surgery are equivalent to those found with mastectomy. 1 2 3 4 5 6

Skin-sparing mastectomy (SSM) and nipple-sparing mastectomy (NSM), traditionally recommended for selected cases in which tumor-to-nipple distance is > 2 cm, also represented a major progress in the management of breast cancer, facilitating immediate breast reconstruction and becoming increasingly popular due to the more satisfactory cosmetic outcome. 7 8 Nevertheless, since no prospective randomized studies have been conducted to evaluate the oncological safety of these techniques, their use in clinical practice is based predominantly on retrospective studies, many of which involve small sample sizes. 9 10 11 12 13 14 Therefore, numerous questions remain regarding expanding the criteria for recommending NSM. Controversial issues include its use in locally advanced cases, the criterion for tumor-to-nipple distance, the routine use of frozen section of the base of the nipple, the need for postmastectomy radiotherapy, and the thickness of the skin flap. 15 16 17 18 19

The primary objective of the present study was to evaluate the oncological outcomes of patients submitted to NSM in an unselected population seen at a single Brazilian institute.

## Methods

This cross-sectional retrospective study evaluated a cohort of unselected patients with invasive carcinoma or ductal carcinoma in situ (DCIS) in whom NSM had been performed between 2010 and 2020. All patients were operated by the same surgical team in an institute working exclusively within Brazil's public healthcare system (SUS, or *Sistema Único de Saúde* ). The institute's internal review board approved the study protocol under reference CAAE: 42697221.8.0000.5040. Since the data were collected retrospectively and anonymously, no specific written informed consent was required.

The recommendation for NSM was made on an outpatient basis following physical examination and imaging evaluation, normally mammography and ultrasonography, with no breast magnetic resonance imaging (MRI) being performed. Neither tumor size nor tumor-to-nipple distance was taken into consideration in the recommendation for NSM irrespective of whether the patient had an invasive carcinoma or DCIS. On imaging evaluation, tumor-to-nipple distance was ≤ 2 cm in all cases. Patients who had previously had breast cancer or had Paget disease of the nipple, and those with metastatic disease at diagnosis were excluded, as were patients who had undergone a free nipple graft and those with incomplete data on their hospital records.

The surgical technique used in NSM was individualized for each patient based on the subcutaneous plane of the breast, and the minimum thickness of the flap was not stipulated. In general, the type of incision in NSM depended on breast size/volume, on ensuring the best possible cosmetic outcome, and on oncological criteria. The skin flap was meticulously dissected using an electric scalpel after the superficial fascia had been identified, according to anatomical and oncological criteria, up to the limits of the breast silhouette, previously identified with the patient in a seated position. Frozen section analysis of the base of the nipple was not routinely performed; however, the nipple margin was always evaluated at histopathology of the surgical specimen, together with all the margins in the specimen. Clear margins were defined as “no ink on tumor” for invasive tumors, and as free margins greater than 2 mm for DCIS. Surgical evaluation of the axilla was carried out through a small separate incision in the axilla for identification of the sentinel lymph node and for performing axillary dissection whenever necessary. The decision regarding the type of immediate breast reconstruction to be performed was made at the discretion of the surgical team. The indication for systemic treatment and for radiotherapy was based on histopathological and immunohistochemical criteria, following the recommendations laid out in the international guidelines. Imaging tests were not used to measure flap thickness postoperatively.

Data such as age, the date of surgery, the duration of follow-up after surgery, and the type of incision (inframammary fold, periareolar, radial or other) were collected. In addition, tumor staging was evaluated according to the eighth edition of the American Joint Committee on Cancer (AJCC) Cancer Staging Handbook: tumor size (T), final lymph node status (N), histological type, and whether the disease was invasive or in situ (low or high-grade). In the case of upfront surgery, T and N status were assessed through histological analysis of the surgical specimen, while in cases in which systemic neoadjuvant therapy was used, the initial stage prior to treatment was the factor taken into consideration. The subtype of tumor in cases of invasive disease was classified according to the results of immunohistochemistry performed on the biopsy specimen: patients expressing hormone receptors and who did not have HER2 overexpression (HER2) were considered hormone-positive or luminal, while patients with HER2 expression were classified as such, irrespective of hormone receptor status, and cases with no hormone receptor expression and no overexpression of HER2 were considered triple-negative. Any systemic treatment received was also evaluated: adjuvant or neoadjuvant chemotherapy, hormone therapy, and radiotherapy.

The oncological outcomes evaluated were local recurrence (recurrence in the reconstructed breast), contralateral recurrence, regional recurrence (including axillary and internal mammary chains), distant recurrence and death. The 5-year disease-free survival (DFS) rate was defined as the proportion of live patients with no signs of locoregional disease, or contralateral or distant metastasis up to the time of the last follow-up. The overall survival (OS) rate was based on the proportion of patients alive at the last follow-up. Kaplan-Meier curves were developed for patients with invasive carcinoma beginning on the date of surgery to evaluate OS and DFS.

## Results

The study population consisted of 76 consecutive patients with invasive or in-situ carcinomas operated on between November 2010 and December 2020. Thirteen of these (17%) were operated on between 2010 and 2015, while 63 (83%) underwent surgery between 2016 and 2020. The mean time of follow-up after NSM was 34.8 months (range 5–114 months). Clinical, surgical, and pathological characteristics are described in Table 1 . The mean age of patients was 46.1 years (27–73 years). The most common incisions used were inframammary fold in 35 cases (46%), periareolar in 34 (40%), and radial in 7 (9%). All patients underwent implant-based breast reconstruction. Twenty-seven patients (36%) were submitted to direct-to-implant reconstruction and 49 (64%) to 2-stage breast reconstruction. Fifty-eight patients had an invasive carcinoma, while 18 had a DCIS. The most common subtype of invasive carcinoma consisted of hormone-positive tumors (60%) followed by HER2 (24%) and triple-negative (16%). Of the cases of DCIS, histological grade was high in 39%.

**Table 1:** Clinical, surgical, and pathological characteristics of the 76 consecutive patients submitted to nipple-sparing mastectomy for breast carcinomas

	Invasive carcinoma		Ductal carcinoma in situ	
	n	%	n	%
Number of cases diagnosed	58		18	
*Grading of ductal carcinoma in situ*				
High grade			7	39
Low grade			11	61
*Subtype of invasive carcinoma*				
Luminal	35	60	−	−
Triple-negative	9	16	−	−
HER2	14	24	−	−
*Type of incision*				
Periareolar incision	23	40	11	61
Radial incision	7	12	0	0
Inframammary fold incision	28	48	7	39
*Tumor size*				
Tis	1	2	18	100
T1	16	28	0	0
T2	38	66	0	0
T3	3	5	0	0
*Axillary status*				
N0	36	62	18	100
N1	13	22	0	0
N2	8	14	0	0
N3	1	2	0	0
*Treatment*				
Neoadjuvant chemotherapy	22	38	0	0
Adjuvant chemotherapy	29	50	0	0
Endocrine therapy	41	71	0	0
Radiotherapy	22	38	1	6

In the majority of cases of invasive carcinoma, tumor size was T2 (66%), followed by T1 (28%), and T3 (5%). Axillary status was positive in 38% of cases and classified as follows: N1 (22%), N2 (14%), and N3 (2%). The resection margins of the surgical specimens were free of disease in all cases. All the patients with invasive carcinomas underwent some form of systemic treatment: chemotherapy (n = 51, 88%), with this consisting of neoadjuvant chemotherapy in 22 women (38%), and hormone therapy in 41 patients (71%). Radiotherapy was performed in 38% of cases of invasive carcinoma. Patients with DCIS underwent no systemic therapy.

**Fig. 1:**
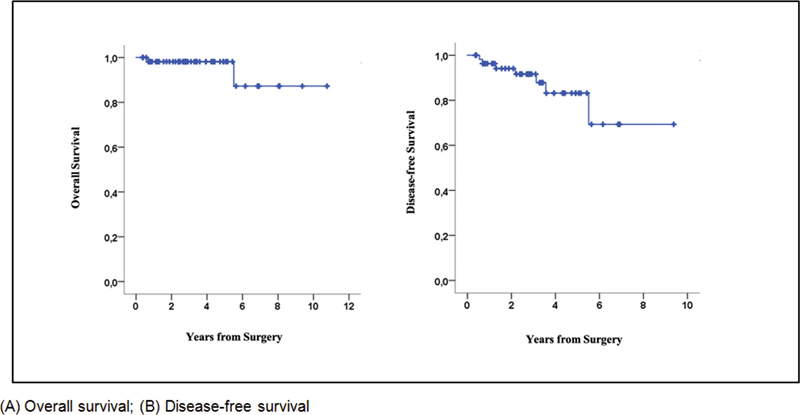
Five-year oncological outcome of nipple-sparing mastectomy in invasive carcinoma.

Four patients had a local recurrence: three cases of invasive carcinoma (5.1%), one of which was in the nipple-areola complex (NAC), and one in a patient with DCIS, also in the NAC (5.5%). In relation to cases of recurrence in the NAC, one patient had invasive HER2 disease (irregular mass; 1.8 cm from the NAC) and the other, a DCIS (microcalcifications; 2 cm from the nipple). Recurrence in the contralateral breast was recorded in two cases. Two patients had distant metastasis and two deaths occurred during the follow-up period ( Table 2 ), all in patients with an invasive carcinoma, resulting in a 5-year OS rate of 98% and a DFS rate of 83% ( Figure 1 ).

**Table 2:** Recurrences following nipple-sparing mastectomy in invasive or in situ carcinomas

	Invasive carcinoma		Ductal carcinoma in situ	
	n	%	n	%
Local recurrence	3	5.1	1	5.5
Regional recurrence	0	0	0	0
Recurrence in the contralateral breast	1	1.7	0	0
Distant metastasis	2	3.4	0	0
Death	2	3.4	0	0

## Discussion

In the present analysis, the OS rate and the local recurrence rate were acceptable and comparable with data from previously published studies, both for invasive and for in-situ disease. Indeed, the rate of local recurrence detected over the study period was around 5%, with a 5-year OS rate of 98%, despite the fact that 71% of the sample of invasive tumors consisted of T2/T3 tumors, and the axilla was positive in 38%, the same proportion of patients who received neoadjuvant chemotherapy. An Italian study involving 1,989 patients reported similar results, with a local recurrence rate of 5.3% in cases of invasive breast cancer and 4% in cases of in-situ disease after a follow-up time of 94 months. Around 50% of that sample had T2/T3 disease or positive axilla. 10

Retrospective studies have shown that NSM is a safe alternative to conventional mastectomy. Although this is a technically more complex surgery, delays in providing adjuvant therapies are uncommon and locoregional control of the disease is generally excellent. 8 10 11 12 20 Most of those studies, however, evaluated selected populations, predominantly at clinical stages I or II. 8 13 21 Expansion of the use of NSM has been a subject of debate; however, the oncological safety of NSM in locally more advanced tumors (stage IIB or higher) or in patients receiving neoadjuvant chemotherapy has not been sufficiently demonstrated. 22 23

Tumor-to-nipple distance is also a topic that has generated much debate. Traditionally, a tumor-to-nipple distance of 2 cm has been suggested as the limit criterion for performing NSM; however, this is not uniformly accepted in the literature. 24 25 26 27 In the present study, the recommendation for performing NSM was not based on any specific tumor-to-nipple distance. The findings of the present study reject the concept of a tumor-to-nipple distance > 2 cm. In fact, our group has expanded the indication of NSM over the years to include patients with locally advanced disease. We believe that tumor-to-nipple distance < 2 cm should not be a contraindication for the technique, particularly in the era of multimodal treatment. This rationale is applied in breast-conserving surgery, in which, paradoxically, this type of surgery has decreased over the years without affecting control of the disease. 1 2 3 4 5 6 We considered that the absence of any signs that the NAC was affected by the disease at clinical evaluation and imaging tests, associated with a final histopathology result showing disease-free margins, would be sufficient. An evaluation of 193 patients with invasive disease and a tumor-to-nipple distance > or < 2 cm, measured using MRI, found no statistically significant difference in terms of DFS. 27 Another study reporting similar results following the analysis of 245 patients submitted to NSM and previous MRI measurements found no statistically significant differences in terms of DFS and survival without local recurrence after 60 months of follow-up irrespective of tumor-to-nipple distance (< or ≥ 2 cm). 26 Furthermore, a recent evaluation comparing the oncological outcome of patients in whom tumor-to-nipple distance was ≤ 1 cm versus a group in which tumor-to-nipple distance was > 1 cm, as measured by imaging tests, detected no statistically significant difference in local recurrence after more than 100 months of follow-up. 25

Other controversial issues concern intraoperative margin assessment and the need for NAC resection if disease-positive. 24 28 29 30 31 In the present study, frozen section examination was not routinely performed, with decisions regarding management being based on the final histological analysis of the surgical specimen. Initially, the possibility of a false-positive result is a motive of concern, particularly in low-grade lesions. 29 Furthermore, identification of disease at frozen section examination does not necessarily mean recurrence in the NAC. Analysis of 948 NSM and 88 false-negatives at frozen section examination showed that the 5-year accumulated rate of local recurrence in the NAC was 2.4%, suggesting that it is possible to preserve the NAC in selected cases following an interdisciplinary debate and discussion with the patient. 31 Another possible strategy in such cases is to remove only the nipple, preserving the rest of the areola. In a study involving 1,326 patients submitted to NSM in which 46 nipple margins were positive, the nipple alone was removed in 51% of cases, and no recurrences were found in the NAC after 36 months of follow-up, suggesting that partial resection of the NAC can represent a safe option. 30

Recently, the debate regarding the appropriate thickness of the NSM flap has intensified. Since this is a technically more complicated procedure and surgical access is more limited, the possibility of leaving residual breast tissue has been controversial. 19 32 33 34 A survey conducted with radiation oncologists and surgeons concluded that the ideal flap within the context of conservative mastectomy (SSM and NSM) would be between 1 and 5 mm. 33 In the present study, no specific cut-off point was stipulated for the thickness of the flap, and the flap was not evaluated with routine postsurgical imaging tests. In our opinion, the thickness of the flap in NSM depends on the anatomical structure of each individual patient and leaving breast tissue behind does not necessarily imply a greater likelihood of recurrence. A review of mastectomies (SSM) found residual breast tissue even in flaps with thickness < 5 mm, showing that “more radical” flaps may not eliminate the risk of residual breast tissue. 15 Furthermore, thinner flaps are known to be associated with a greater likelihood of ischemic complications, suggesting a need to individualize the technique among women. 32 Using presurgical MRI measurements, a study evaluated the flap thickness in NSM as a function of body mass index (BMI) categories. 33 The findings showed that BMI < 25, 25–30 and > 30, as well the weight categories of the mastectomy (< 400 g, 400–799 g and > 800 g), were associated with flap thicknesses, with each increase being statistically significant.

Another related topic is the need for radiotherapy. Many argue that if NSM constitutes conservative surgery, then the patient is a natural candidate for radiotherapy. However, various authors have questioned the need for radiotherapy in NSM due to the low rate of local recurrence found: in a study conducted in Italy, radiotherapy of the whole thoracic wall was given in 6.7% of cases following NSM, with local recurrence reported in around 5% of the women, although the axillary lymph nodes were affected in around 50% of the patients. 10 Paradoxically, patients submitted to NSM still appear more likely to receive radiotherapy compared to those submitted to less conservative mastectomies. A review conducted using the Surveillance, Epidemiology, and End Results (SEER) program database identified 470 patients who had undergone NSM between 2006 and 2010 and 112,347 who had not, with results showing that the women in the NSM group were more likely to have undergone radiotherapy. 35

There are some limitations associated with the present study. Since this was a retrospective cohort design, and the study was conducted in one single institute, there is a possibility that biases could have affected the findings. The small number of events could also have affected the analysis, as well as the short follow-up time. On the other hand, the strongpoint of this study lies in the fact that the population analyzed was unselected, and healthcare was provided within the public sector.

## Conclusion

The present findings corroborate those of other retrospective series on NSM, with a low rate of local recurrence, even in an unselected population, and an excellent OS rate. Nevertheless, further studies are required to improve understanding of this approach.
